# High Prevalence of Cysticercosis in People with Epilepsy in Southern Rwanda

**DOI:** 10.1371/journal.pntd.0002558

**Published:** 2013-11-14

**Authors:** Ruth Rottbeck, Jules Fidèle Nshimiyimana, Pierrot Tugirimana, Uta E. Düll, Janko Sattler, Jean-Claudien Hategekimana, Janvier Hitayezu, Irmengard Bruckmaier, Matthias Borchert, Jean Bosco Gahutu, Sebastian Dieckmann, Gundel Harms, Frank P. Mockenhaupt, Ralf Ignatius

**Affiliations:** 1 Department of Internal Medicine/Neurology, Butare University Teaching Hospital, Butare, Rwanda; 2 Gikonko Health Center, Gikonko, Rwanda; 3 Institute of Tropical Medicine and International Health, Charité – Universitätsmedizin Berlin, Berlin, Germany; 4 Clinical Department, Medical Biology, Butare University Teaching Hospital, Butare, Rwanda; University of Oklahoma Health Sciences Center, United States of America

## Abstract

**Background:**

Neurocysticercosis (NCC), the central nervous system infection by *Taenia solium* larvae, is a preventable and treatable cause of epilepsy. In Sub-Saharan Africa, the role of NCC in epilepsy differs geographically and, overall, is poorly defined. We aimed at contributing specific, first data for Rwanda, assessing factors associated with NCC, and evaluating a real-time PCR assay to diagnose NCC in cerebrospinal fluid (CSF).

**Methodology/Principal findings:**

At three healthcare facilities in southern Rwanda, 215 people with epilepsy (PWE) and 51 controls were clinically examined, interviewed, and tested by immunoblot for cysticerci-specific serum antibodies. Additionally, CSF samples from PWE were tested for anticysticercal antibodies by ELISA and for parasite DNA by PCR. Cranial computer tomography (CT) scans were available for 12.1% of PWE with additional symptoms suggestive of NCC. The Del Brutto criteria were applied for NCC diagnosis. Cysticerci-specific serum antibodies were found in 21.8% of PWE and 4% of controls (odds ratio (OR), 6.69; 95% confidence interval (95%CI), 1.6–58.7). Seropositivity was associated with age and lack of safe drinking water. Fifty (23.3%) PWE were considered NCC cases (definitive, based on CT scans, 7.4%; probable, mainly based on positive immunoblots, 15.8%). In CSF samples from NCC cases, anticysticercal antibodies were detected in 10% (definitive cases, 25%) and parasite DNA in 16% (definitive cases, 44%). Immunoblot-positive PWE were older (medians, 30 *vs.* 22 years), more frequently had late-onset epilepsy (at age >25 years; 43.5% *vs.* 8.5%; OR, 8.30; 95%CI, 3.5–20.0), and suffered from significantly fewer episodes of seizures in the preceding six months than immunoblot-negative PWE.

**Conclusions/Significance:**

NCC is present and contributes to epilepsy in southern Rwanda. Systematic investigations into porcine and human cysticercosis as well as health education and hygiene measures for *T. solium* control are needed. PCR might provide an additional, highly specific tool in NCC diagnosis.

## Author Summary

Neurocysticercosis (NCC) is the infection of the brain with larvae of the pig tapeworm (*Taenia solium*), which results from the ingestion of, e.g., food or water contaminated with the eggs of this helminth. Seizures and epilepsy are the most often reported clinical manifestations. The diagnosis is based on various clinical, radiological, microbiological, and epidemiological criteria. NCC is treatable and preventable. Knowledge about its contribution to epilepsy in a given region therefore helps to manage and prevent the disease. Our study aimed at investigating the prevalence of and risk factors associated with NCC in southern Rwanda. Additionally, we evaluated a new diagnostic method based on the detection of parasite DNA in the cerebrospinal fluid of people with epilepsy for its usefulness. By applying well-established diagnostic criteria for NCC, we identified 16 definitive and 34 probable NCC cases among 215 people with epilepsy in southern Rwanda. The risk of NCC was higher in those lacking access to safe drinking water. This highlights opportunities for the prevention of NCC, and consequently, epilepsy in this region. Parasite DNA could be detected in the cerebrospinal fluid of 16% of the NCC patients. Thus, this method might help to identify NCC cases, particularly when radiology cannot be performed easily.

## Introduction

Epilepsy is a common chronic neurological disorder with a median prevalence of 15 in 1000 in sub-saharan Africa [1], [2], and from 7 to 49 in 1000 in Rwanda [3], [4]. Studies on the etiology of epilepsy in the tropics have revealed the importance of central nervous system (CNS) infections, such as neurocysticercosis (NCC), hydatidosis, tuberculomas, cerebral malaria, meningoencephalitis, and HIV/AIDS related opportunistic infections, e.g., cryptococcosis or toxoplasmosis [5]–[7].

NCC is the most severe manifestation of cysticercosis, i.e., the infection with the larval stages of *Taenia solium* [8], and besides the brain, other organs, e.g., skin and muscle, can also be affected by cysticerci. Man is the definitive host of adult *T. solium* tapeworms in the intestine following infection by larvae in undercooked pork. In contrast, cysticercosis results from ingestion of *T. solium* eggs through contaminated food, water, or the environment, or from direct contact with and accidental ingestion of feces of tapeworm carriers including autoinfection. Seizures and epilepsy are the most common manifestation of NCC occurring in approximately 80% of patients [9].

NCC is considered a major public health problem in Africa [10]–[12], but data are sparse for many countries including Rwanda. We therefore aimed at estimating the proportion of PWE suffering from NCC in southern Rwanda and at assessing associated factors. Today, the diagnosis of NCC commonly rests upon the Del Brutto criteria [13], [14]. These are based on radiological imaging and the detection of anticysticercal antibodies in serum by immunoblot or in cerebrospinal fluid (CSF) by ELISA, and on clinical and epidemiological data. Detection of anticysticercal antibodies by immunoblot is considered the serological gold-standard [15]. PCR techniques, however, may offer additional possibilities in NCC diagnosis [16]–[18], and the analysis of stool samples by PCR has recently been shown to be highly sensitive and specific for the identification of taeniasis patients (who are not necessarily cysticercosis or NCC patients as well) [19]. We therefore additionally evaluated the usefulness of a previously described real-time PCR assay for the detection of *T. solium*-specific DNA in CSF in defining NCC cases.

## Materials and Methods

### Study sites and study population

We conducted a health-facility based study in the southern province of Rwanda from February to April 2010. A total of 215 people with epilepsy (PWE) were consecutively recruited at Gikonko Health Center (*n* = 85), Kabutare District Hospital (*n* = 87), and Butare University Teaching Hospital (*n* = 43). These numbers correspond to the PWE who were willing to participate during the three months of recruitment (very few PWE only declined but were not documented); the PWE study participants thus represent a convenience sample. Most of the PWE were on regular treatment for epilepsy and presented for their regular follow-up check at the health facility, but newly diagnosed PWE also were included. Neuroimaging using computer tomography (CT) could not be done as part of this study because it was not available at any recruitment site at the time of the study. However, 26 PWE (12.1%) had had CT scans taken within the last two years prior to the inclusion in the study; these patients had presented with additional symptoms, e.g., headache or hemiparesis, or clinical signs indicative of NCC, e.g., subcutaneous cysts, and could afford a CT scan. Epilepsy was defined as two or more unexplained, unprovoked seizures [20]. The criteria of Sander and Shorvon [21] for tonic-clonic seizures were applied. Inclusion criteria for cases were i) informed written consent, ii) age ≥10 years, iii) residence in the southern Province of Rwanda, and iv) diagnosis of epilepsy. Fifty-one patients (Gikonko Health Center, 15; Kabutare District Hospital, 13; Butare University Teaching Hospital, 23) presenting for general surgical problems (e.g., fractures, wounds) but otherwise healthy were chosen as control group. This number represents a convenience sample of all patients admitted for general surgery to the three health facilities within the study period and who agreed to participate in the study. None of the controls had a history of convulsions or epilepsy, which would have been an exclusion criterion.

### Data for PWE and controls

An extensive questionnaire on demographic background, family history, own medical history, risk factors for epilepsy, and cysticercosis was filled in for all study participants. Ongoing treatment for epilepsy was recorded as well as previous treatment for cysticercosis or *T. solium* taeniasis. A physical examination (a complete neurological examination including mental state, muscle tonus and weakness, pyramidal signs etc. as well as an examination for subcutaneous or lingual cysts) was done in all study participants and additionally funduscopy in 205 PWE. The cysticercal origin of subcutaneous cysts was proven by scolex detection through ultrasound. Information on previous brain CT scans and electroencephalography (EEG) reports was obtained by asking the PWE or a relative or consulting the files of the PWE at the respective health facility. An EEG report was available for 27 PWE (12.6%, normal, 5; generalized slowing, 10; focus and/or epileptic activity, 12).

### Laboratory tests

Venous blood was collected from all participants, and serum stored at −20°C. Serum samples were subjected to an HIV antibody ELISA (Vironostika, bioMérieux, Boxtel, The Netherlands) and positive results were confirmed with a second ELISA test (Murex, Abbott, Rungis Cedex, France). Stool samples were collected from all PWE and examined microscopically (following concentration techniques) for helminth ova [22].

At the same time as the blood samples, CSF was collected by lumbar puncture from 214 PWE (if signs of raised intracranial pressure, e.g., decreased level of consciousness/confusion, papilledemia, or a focal neurological deficit were present, a brain CT scan was performed at the King Faysal Hospital in Kigali, which is 125 km away from Butare, before lumbar puncture). CSF samples were stored at 4°C and transported within a maximum of five hours to the laboratory of the Butare University Teaching Hospital. CSF anti-cysticercal antibodies were determined by ELISA (DRG Diagnostics, Marburg, Germany), and remaining CSF was stored at −20°C. CSF and serum samples were transported on dry ice to the Institute of Tropical Medicine and International Health, Berlin. There, serum samples were analyzed by immunoblot (Immunetics, Boston, MA, USA; sensitivity, 95%, specificity, 100% in cysticercosis patients; information provided by the manufacturer) for the presence of anticysticercal antibodies.

Real-time PCR was performed for all CSF samples as described previously [18] except that we used twice as much specimen as published, i.e., 200 µl instead of 100 µl. Briefly, DNA was extracted by commercial kits (Qiamp DNA Blood Mini Kit, Qiagen), and assays were run on a Lightcycler 480 (Roche Diagnostics). A cycle threshold (Ct) value of >42 was considered to reflect limited reproducibility due to low copy numbers, and all respective assays were repeated. In addition, for all NCC cases defined by using the Del Brutto criteria, PCR assays were repeated using DNA extracted from the total volume of CSF (1.2–1.8 ml).

### Diagnosis of NCC

Criteria used for the diagnosis of NCC were i) ***absolute***, e.g., visualization of a scolex by CT scan (CT scans were available for 26 PWE with additional symptoms suggestive of NCC) or of subretinal parasites via funduscopy, ii) ***major***, e.g., positive serum immunoblot or highly suggestive lesions on CT, iii) ***minor***, e.g., lesions compatible with NCC on CT, typical clinical manifestations, positive CSF ELISA, or cysticercosis outside of the CNS, and iv) ***epidemiological*** criteria, e.g., household contact with *T. solium* infection or living in an endemic area. A definitive diagnosis is given by the presence of one absolute or two major plus one minor and one epidemiological criteria, a probable diagnosis is justified when i) one major plus two minor criteria, ii) one major plus one minor and one epidemiological criteria, or iii) three minor plus one epidemiological criteria are present [14]. Due to the lack of CT scans for most PWE, the presence of a positive serum immunoblot plus typical symptoms plus epidemiology allowed the identification of probable NCC cases only.

### Ethics statement

All PWE and controls (and parents/custodians if applicable) were thoroughly informed about the purpose and procedures of the study, and recruitment was preceded by obtaining informed written consent from participants or parents/custodians. When participants or parents/custodians were unable to read, the participant information was read and explained to them by the physician in the presence of a relative or attendant, and the participant used his or her thumb to sign. The study was reviewed and approved by the National Ethics Committee, Republic of Rwanda.

### Statistical analysis

Data analysis was performed using Statview 5.0 (SAS Institute Inc.). The numbers of individuals analyzed are presented and may vary due to missing data. Continuous variables were compared between groups by the Mann-Whitney or Kruskal-Wallis test, and proportions by χ^2^ test or Fisher's exact test. Prevalence odds ratios (ORs) and 95% confidence intervals (95% CI) were calculated. A *P*-value <0.05 was considered statistically significant. For the outcome epilepsy, we entered all factors found to be associated (*P*<0.10) with the outcome in bivariable analysis into a logistic regression model. We manually identified and maintained those variables as confounders that changed the odds ratio between the primary exposure “immunoblot-positive” and the outcome “epilepsy” substantially. We furthermore manually identified and maintained those variables as secondary exposures that were found to significantly improve the goodness of fit of the model (likelihood ratio test, *P*<0.05). For the outcome “immmunoblot-positive”, we applied an explorative, i.e. not hypothesis-driven analysis of factors associated with seropositivity by immunoblot. All factors found to be associated (*P*<0.10) with the outcome in bivariable analysis were entered into a logistic regression model and maintained if they significantly improved the goodness of fit of the model (likelihood ratio test, *P*<0.05) in manual backward reduction. For the comparison of diagnostic tests, the positive and negative percent agreement was calculated.

## Results

### Epidemiological and clinical characteristics of PWE and controls

The socio-demographic and clinical characteristics of 266 individuals studied (215 PWE, 51 controls) are shown in Table S1. Most study participants were residents of Gisagara and Huye districts, had been living there since birth, and lacked formal education or had attended primary school only. PWE were of roughly half the age of controls, and comparably more were male. As compared to controls, more PWE had a family history for epilepsy (28% vs. 11.7%, *P* = 0.02), and more remembered previous head injuries (16% vs. 4%, *P* = 0.02). Fourteen percent of the PWE presented a mental retardation (according to the clinical impression of the interviewing physician). Neurological examination did not reveal further significant differences between PWE and controls. Notably, more PWE (22%) than controls (4%) had cysticerci-specific antibodies determined by immunoblot (OR, 6.69; 95%CI, 1.63–58.67). More PWE than controls tended to keep pigs at home. No significant differences were seen with respect to pork consumption, access to safe water, defecation habits, or use of fertilizers (Table S1).

### Factors associated with epilepsy

Available data on socio-demographic background, family history, and medical history were tested for association with epilepsy. In univariate analysis, epilepsy was associated with recruitment site and residence (Table 1). In addition, factors associated with reduced odds of epilepsy included increasing age, female sex, and living at the current location for more than ten years but not been born there. A family history of epilepsy was positively associated with epilepsy. For serologically anti-cysticerci antibody positive individuals, the odds of epilepsy were increased almost seven-fold (OR, 6.7; 95%CI, 1.6–58.7).

**Table 1:** Odds ratios and adjusted odds ratios (95% confidence intervals) for epilepsy.

Variable	No.	PWE^a^	Controls^a^	OR	95%CI	*P*	aOR^b^	95%CI	*P*
Age (years)	266	23 (10–65)	40 (15–73)	0.92	0.90–0.94	**<0.0001**	0.90	0.87–0.94	**<0.0001**
Sex									
Male	140	55.8 (120/215)	39.2 (20/51)	1					
Female	126	44.2 (95/215)	60.8 (31/51)	0.51	0.26–0.99	**0.03**	0.27	0.10–0.73	**0.009**
Location of recruitment									
Butare, university hospital	66	20.0 (43/215)	45.1 (23/51)	1					
Kabutare, district hospital	100	40.5 (87/215)	25.5 (13/51)	3.58	1.55–8.34	**0.0008**	1.98	0.67–5.91	0.22
Gikonko, health center	100	39.5 (85/215)	29.4 (15/51)	3.03	1.35–6.85	**0.003**	6.10	1.54–24.18	**0.01**
Residence, district									
Gisagara	103	39.1 (84/215)	37.3 (19/51)	1					
Huye	119	47.0 (101/215)	35.3 (18/51)	1.27	0.59–2.72	0.51	2.00	0.59–6.72	0.26
Nyamagabe	3	0.9 (2/215)	2.0 (1/51)	0.45	0.02–28.1	0.52	0.12	0.00–3.44	0.22
Nyanza	18	7.0 (15/215)	5.9 (3/51)	1.13	0.28–6.69	1.0	1.36	0.16–11.79	0.78
Nyaruguru	13	4.2 (9/215)	7.8 (4/51)	0.51	0.13–2.52	0.29	1.24	0.16–9.53	0.83
Others	10	1.9 (4/215)	11.8 (6/51)	0.15	0.03–0.72	**0.008**	0.18	0.03–1.32	0.09
Residence at current location since (%)^c^									
Since birth	230	88.3 (189/214)	80.4 (41/51)	1					
>10 years^d^	13	2.8 (6/214)	13.7 (7/51)	0.19	0.05–0.69	**0.005**	0.27	0.06–1.30	0.10
2–10 years^d^	15	6.1 (13/214)	3.9 (2/51)	1.41	0.30–13.3	1.0	1.25	0.21–7.51	0.81
<2 years^d^	7	2.8 (6/214)	2.0 (1/51)	1.30	0.15–61.3	1.0	0.88	0.04–20.19	0.94
Family history of epilepsy (%)									
None	200	72.1 (155/215)	88.2 (45/51)	1					
First degree relative(s)	32	14.0 (30/215)	3.9 (2/51)	4.35	1.03–38.9	**0.03**	9.31	1.12–77.13	**0.04**
Other relative(s)	34	14.0 (30/215)	7.8 (4/51)	2.18	0.71–8.93	0.15	14.11	2.04–97.57	**0.007**
Cysticercosis immunoblot^e^									
Negative	213	78.2 (165/211)	96.0 (48/50)	1					
Positive	48	21.8 (46/211)	4.0 (2/50)	6.69	1.63–58.67	**0.003**	26.98^f^	3.79–192.12	**0.001**

a , data are medians (range) for age and proportions (%, n/n) among PWE and controls, respectively;

b , adjusted odds ratios originate from a logistic regression model including all shown variables, n = 260, correlation coefficient R^2^ = 0.44;

c , data for one person with epilepsy missing;

d , born elsewhere;

e , data for four PWE and one control missing;

f , removing the variable “Residence, district” from the model would lead to an aOR of 30.96 (95%CI, 4.52–212.22) for the cycticercosis immunoblot; removing the variable “Residence at current location since”, the aOR would change to 32.39 (95%CI, 4.48–234.15); removing both, the aOR would turn 36.05 (95%CI, 5.16–252.06).

In multivariate analysis, residence and the time spent there since birth lost significance, and the associations of epilepsy with family history and immunoblot result were strengthened. In particular, being seropositive for cysticerci-specific antibodies greatly increased the odds of epilepsy (adjusted OR, 26.98; 95%CI, 3.79–192.12; Table 1).

### Factors associated with a positive serum immunoblot result

Since a positive immunoblot result was strongly linked with epilepsy, we next looked into factors associated with the presence of cysticerci-specific antibodies among PWE. As before, data from socio-demographic background, family history, and medical history were tested for association. Since both variables, “Residence, district” and “Residence at current location since”, were identified as confounders of the association between EITB and being PWE (aORs given in footnote of Table 1), both were maintained in the model, although the likelihood ratio test suggested that they did not significantly improve the model's goodness of fit. Factors positively associated in univariate analysis included increasing age, previous albendazole treatment, and pork consumption (Table 2). In contrast, antibody responses were less likely in individuals from the primary health center. These univariate associations remained significant in multivariate analysis (Table 2) except for pork consumption (adjusted OR, 2.60; 95%CI, 0.77–8.71). In addition, in multivariate analysis, lack of safe water supply increased the odds of being immunoblot positive four-fold (Table 2).

**Table 2:** Odds ratios and adjusted odds ratios (95% confidence intervals) for a positive immunoblot result in PWE.

Variable	No.	Immunoblot- positive^a^	Immunoblot- negative^a^	OR	95%CI	*P*	aOR^b^	95%CI	*P*
Age (years)	211	30 (10–65)	22 (10–62)	1.07	1.04–1.10	**<0.0001**	1.07	1.04–1.10	**<0.0001**
Location of recruitment									
Butare, university hospital	43	30.4 (14/46)	17.6 (29/165)	1					
Kabutare, district hospital	83	47.8 (22/46)	37.0 (61/165)	0.75	0.31–1.80	0.48	1.00	0.39–2.55	0.99
Gikonko, health center	85	21.7 (10/46)	45.5 (75/165)	0.28	0.10–0.76	**0.004**	0.31	0.11–0.85	**0.02**
Previous albendazole treatment									
No	176	69.6 (32/46)	87.3 (144/165)	1					
Yes	35	30.4 (14/46)	12.7 (21/165)	3.00	1.29–6.98	**0.004**	2.95	1.18–7.35	**0.02**
Pork consumption									
No	42	8.7 (4/46)	23.0 (38/165)	1					
Yes	169	91.3 (42/46)	77.0 (127/165)	3.14	1.04–12.78	**0.03**			
Safe water supply									
Yes	31	6.5 (3/46)	17.0 (28/165)	1					
No	180	94.5 (43/46)	83.0 (137/165)	2.93	0.84–15.73	0.08	4.27	1.13–16.16	**0.03**

a , data are medians (range) for age and proportions (%, n/n) among PWE with and without a positive immunoblot;

b , adjusted odds ratios originate from a logistic regression model including all shown variables, n = 211, correlation coefficient R^2^ = 0.17.

### Clinical and diagnostic characteristics of PWE with a positive serum immunoblot result

Of 211 PWE tested, 21.8% were seropositive for cysticerci-specific antibodies (i.e., probable NCC, see below). As compared to seronegative PWE, immunoblot-positive PWE were older (Table 2), the age at the first seizure was higher [median (range), 23.5 (1–63) *vs.* 9.0 (0–47) years; *P*<0.0001], late onset epilepsy (defined as age of first seizure >25 years) consequently was more common, and they had fewer epileptic episodes (Table 3). Adjusting for age, the odds of late onset epilepsy was six-fold increased in immunoblot-positive as compared to immunoblot-negative PWE (adjusted OR, 6.0; 95%CI, 1.99–18.09), and the odds of ≥1 episodes of seizure per day tended to be more than halved (adjusted OR, 0.42; 95%CI, 0.17–1.03). All four PWE with impaired visual acuity had a positive immunoblot but their funduscopy did not reveal signs of intracranial pressure or subretinal cysts. Anti-epileptic medication differed slightly between the two PWE groups. Differences in available CT scans are detailed in Table S2. All PWE with subcutaneous cysts or anticysticercal antibodies in CSF samples had positive immunoblot results.

**Table 3:** Selected characteristics of PWE with NCC (defined by a positive serum immunoblot result).

Variable	Immunoblot-negative PWE	Immunoblot-positive PWE	Difference in proportions (%)	95%CI	*P*
No.	165	46			
Female sex (%)	76 (46.1)	17 (37.0)	−9.1	−23.7–7.1	0.27
Age at first seizures (years; median, range)	9.0 (0–47)	23.5 (1–63)			**<0.0001**
Late onset epilepsy (>25 years of age) (%)	14 (8.5)	20 (43.5)	35.0	20.7–50.0	**<0.0001**
>1 episode/day of seizures during 6 months (%)	58/161 (36.0)	7 (15.2)	−20.8	−31.9–−6.0	**0.007**
Sphincter disturbance (%)	101/161 (62.7)	20/45 (44.4)	−18.3	−33.5–−2.0	**0.03**
Impaired visual acuity, visual field (%)	0	4 (8.7)	8.7	3.0–20.3	**0.002**
Current treatment (%)					
Phenobarbital	56/161 (34.8)	20 (43.5)	8.7	−6.6–24.6	0.28
Carbamazepine	51/161 (31.7)	12 (26.1)	−5.6	−18.5–10.1	0.47
Valproic acid	19/161 (11.8)	8 (17.4)	5.6	−4.6–19.5	0.32
Phenytoine	0	1 (2.2)	2.2	−0.8–11.3	0.22
Phenobarbital+Carbamazepine	22/161 (13.7)	3 (6.5)	−7.2	−14.6–4.7	0.09
Other	9/161 (5.6)	0	−5.6	−10.3–2.6	0.21
None	4/161 (2.5)	2 (4.3)	1.8	−3.0–12.2	0.62
CT result (%)					
Not available	155 (93.9)	31 (67.4)	−26.5	−41.2–−13.9	**<0.0001**
Normal	5 (3.0)	0	−3.0	−6.9–4.9	0.59
Cystic scolex	1 (0.6)	14 (30.4)	29.8	18.2–44.2	**<0.0001**
Suggestive*	2 (1.2)	1 (2.2)	1.0	−2.6–10.2	0.52
Compatible#	1 (0.6)	0	−0.6	−3.4–7.1	1.0
Atrophic brain	1 (0.6)	0	−0.6	−3.4–7.1	1.0
Subcutaneous cysts (%)	0	9 (19.6)	19.6	10.4–33.2	**<0.0001**
CSF ELISA positive (%)	0/164	5 (10.9)	10.9	4.3–23.0	**0.0004**

* cystic lesion without scolex, single or multiple ring or nodular enhancing lesion, or parenchymal round calcification [14], details given in Table S1.

# hydrocephalus or abnormal enhancement of the leptomeninges [14].

### Definition of NCC cases according to the Del Brutto criteria

CT scan results were available for 26 PWE (12.1%) with additional symptoms suggestive of NCC. Of these, 16 showed cystic lesions with scolex, and three presented lesions highly suggestive of NCC (the details of the neuroimaging results are shown in Table S2). Five scans were normal, one scan showed lesions compatible with NCC, and one atrophy of the brain. Funduscopy done in 205 PWE did not reveal pathological findings.

However, immunoblot results (major Del Brutto criterion, if positive) allowed the definition of probable NCC in the additional presence of two minor criteria or one minor and one epidemiological criterion [14]. Consequently, 50 NCC cases (23.3%) were identified among the 215 PWE (16 definitive, 7.4%; 34 probable, 15.8%; Table 4). Of these, 94% (46/49) had a positive immunoblot and 10% (5/50) a positive CSF ELISA result. The serum sample from the person with epilepsy with documented cerebral lesions compatible with NCC (a minor Del Brutto criterion only) yielded a negative result by the immunoblot. Due to the lack of any major and further minor criteria this individual was not considered a case of NCC.

**Table 4:** Diagnostic data for all PWE with NCC according to the Del Brutto criteria.

ID	CT scan	Immunoblot	CSF ELISA	Skin	Del Brutto	CSF PCR
B005P	Cystic scolex	+	+	Cysts	Definitive	+
B006P	Cystic scolex	+	−	−	Definitive	+
B011P	Suggestive‡	−	−	−	Probable	−
B014P	Suggestive	+	−	−	Probable	−
B016P	Cystic scolex	+	+	Cysts	Definitive	(+)*
B017P	Suggestive	−	−	−	Probable	−
B018P	Cystic scolex	+	−	−	Definitive	−
B019P	Cystic scolex	+	−	−	Definitive	+
B021P	Cystic scolex	−	−	−	Definitive	−
B026P	Cystic scolex	+	−	Cysts	Definitive	−
B032P	Cystic scolex	+	−	−	Definitive	−
B034P	Cystic scolex	+	+	Cysts	Definitive	+
B035P	Cystic scolex	+	−	Cysts	Definitive	(+)
B040P	Cystic scolex	+	−	−	Definitive	−
B041P	Cystic scolex	+	−	−	Definitive	−
B042P	Cystic scolex	+	−	−	Definitive	−
B043P	Cystic scolex	+	−	−	Definitive	−
G006P	n.d.	+	−	−	Probable	−
G019P	n.d.	+	−	−	Probable	−
G021P	n.d.	+	−	−	Probable	−
G027P	Cystic scolex	+	+	Cysts	Definitive	+
G032P	n.d.	+	−	−	Probable	−
G038P	n.d.	+	−	−	Probable	−
G042P	n.d.	+	−	−	Probable	−
G055P	n.d.	+	+	−	Probable	−
G065P	n.d.	+	−	−	Probable	−
G069P	n.d.	+	−	−	Probable	−
K001P	n.d.	+	−	−	Probable	−
K003P	n.d.	+	−	−	Probable	−
K007P	n.d.	+	−	−	Probable	No CSF
K008P	n.d.	+	−	−	Probable	−
K010P	n.d.	+	−	−	Probable	−
K013P	n.d.	+	−	−	Probable	−
K014P	n.d.	+	−	Cysts	Probable	−
K015P	n.d.	+	−	−	Probable	−
K027P	n.d.	+	−	−	Probable	−
K028P	n.d.	+	−	−	Probable	−
K030P	n.d.	+	−	−	Probable	−
K040P	n.d.	+	−	Cysts	Probable	(+)
K045P	n.d.	+	−	−	Probable	−
K053P	n.d.	+	−	−	Probable	−
K055P	n.d.	+	−	−	Probable	−
K057P	n.d.	+	−	Cysts	Probable	−
K060P	n.d.	+	−	−	Probable	−
K064P	n.d.	+	−	−	Probable	−
K067P	n.d.	+	−	−	Probable	−#
K069P	n.d.	+	−	−	Probable	−
K074P	n.d.	+	−	−	Probable	−
K075P	n.d.	+	−	−	Probable	−
K087P	Cystic scolex	n.a.	−	−	Definitive	−

‡ cystic lesion without scolex, single or multiple ring or nodular enhancing lesion, or parenchymal round calcification [14], details given in Table S1.

(+)* , positive only with the maximum CSF volume (1.2–1.8 ml).

# only 0.2 ml CSF for one PCR available.

n.a., no serum sample available.

Notably, the person with epilepsy, B014P (Table 4), had “suggestive lesions” and a positive immunoblot. If he had had a negative serology at the time of the scan, he should have been classified “probable case” at that time due to the presence of one major criterion plus one minor and one epidemiological one. At the time of our study, with the presence of two major criteria, i.e., suggestive lesion and positive immunoblot, plus one minor and one epidemiological criterion, this individual could have been classified as “definitive case” according to the Del Brutto criteria. Due to the uncertainty regarding the individual's serostatus at the time of the CT scan we still classified this individual as “probable case”.

Diagnostic criteria showed considerable overlap: serum samples from all nine PWE with subcutaneous cysts and from all five PWE with positive CSF ELISA yielded positive immunoblot results. Four of the five PWE with positive CSF ELISA results also had subcutaneous cysts and a positive CT scan. *Taenia* spp. eggs were found in two (immunoblot-positive) PWE only.

Three NCC PWE had a negative immunoblot result. They were defined as NCC cases by suggestive lesions in the CT scans *plus* typical symptoms *plus* living in an endemic area (probable cases, n = 2), or the detection of a scolex (definitive case, n = 1).

We next looked at the data separated for PWE for whom CT scans were available (n = 19) and those without scans (n = 31). Of the PWE with CT scan available, 31.6% (6/19; 95%CI, 15.2–54.2%) also had subcutaneous cysts (Table 5) while these were seen in only 9.7% (3/31; 95%CI, 2.6–25.7%) of PWE without CT scan (Table 5). This is not unexpected as PWE with additional symptoms suggestive of NCC, such as subcutaneous cysts, were advised to go to Kigali to receive a CT scan. A similarly uneven distribution was observed regarding positive ELISA results, i.e., 21.1% (4/19; 95%CI, 8.0–43.9%) of the PWE with CT scans also had anti-cysticercal antibodies in their CSF samples whereas these were detected in only one person with epilepsy (3.2%; 95%CI, <0.0001–17.6%) without CT scan.

**Table 5:** Diagnostic data for PWE with (definitive or probable) NCC for whom CT scans were available (n = 19).

ID	CT scan	Immunoblot	CSF ELISA	Skin	Del Brutto	CSF PCR
B005P	Cystic scolex	+	+	Cysts	Definitive	+
B006P	Cystic scolex	+	−	−	Definitive	+
B011P	Suggestive‡	−	−	−	Probable	−
B014P	Suggestive	+	−	−	Probable	−
B016P	Cystic scolex	+	+	Cysts	Definitive	(+)*
B017P	Suggestive	−	−	−	Probable	−
B018P	Cystic scolex	+	−	−	Definitive	−
B019P	Cystic scolex	+	−	−	Definitive	+
B021P	Cystic scolex	−	−	−	Definitive	−
B026P	Cystic scolex	+	−	Cysts	Definitive	−
B032P	Cystic scolex	+	−	−	Definitive	−
B034P	Cystic scolex	+	+	Cysts	Definitive	+
B035P	Cystic scolex	+	−	Cysts	Definitive	(+)
B040P	Cystic scolex	+	−	−	Definitive	−
B041P	Cystic scolex	+	−	−	Definitive	−
B042P	Cystic scolex	+	−	−	Definitive	−
B043P	Cystic scolex	+	−	−	Definitive	−
G027P	Cystic scolex	+	+	Cysts	Definitive	+
K087P	Cystic scolex	n.a.	−	−	Definitive	−

‡ cystic lesion without scolex, single or multiple ring or nodular enhancing lesion, or parenchymal round calcification [14], details given in Table S1.

(+)* , positive only with the maximum CSF volume (1.2–1.8 ml).

n.a., no serum sample available.

### Detection of *T. solium* DNA by real-time PCR

We analyzed the CSF samples from the PWE by *T. solium*-specific real-time PCR using twice as much CSF for DNA extraction as suggested (200 µl instead of 100 µl). Only five of 214 (2.3%) specimens yielded a positive result and three of these were also positive for anti-cysticercal antibodies determined by ELISA (Table 4). Because of the large number of negative results, a different PCR assay was performed that verified the presence of human DNA in 192/214 CSF samples and excluded the possibility of major PCR inhibition. We then used the maximum volume (1.2–1.8 ml) of 48 available CSF samples from the 50 NCC patients for DNA extraction and repeated the *T. solium*-specific PCR assay. Thereby, we detected three additional PCR-positive samples, two from “definitive NCC cases” defined by CT scan and one from a “probable NCC case” with subcutaneous cyst for whom no CT scan was available (Table 4). Thus, PCR detected *T. solium*-specific DNA in CSF samples from 7/16 (43.8%) definitive NCC cases. Notably, four of the eight in total PCR-positive CSF specimens contained anti-cysticercal antibodies.

Seven of the eight PCR positive samples were obtained from PWE who had received a CT scan (7/19, 36.8%; 95%CI, 19.1–59.1%; Table 5). Among these seven “PCR-positive PWE”, five also presented with subcutaneous cysts, i.e., only one sample from the PWE with CT scan and subcutaneous cysts was PCR-negative (Table 5). In contrast, only one CSF sample from the PWE without CT scan who were classified as “probable NCC cases” due to positive immunoblot results, clinical manifestation, and epidemiology yielded a positive PCR result (3.2%; 95%CI, <0.0001–17.6%; Table 6). This sample was obtained from one of the two PWE in this group with subcutaneous cysts.

**Table 6:** Diagnostic data for PWE for whom no CT scans were available and who were classified “probable NCC cases” due to positive immunoblot results, clinical manifestation, and epidemiology (n = 31).

ID	CSF ELISA	Skin	CSF PCR
G006P	−	−	−
G019P	−	−	−
G021P	−	−	−
G032P	−	−	−
G038P	−	−	−
G042P	−	−	−
G055P	+	−	−
G065P	−	−	−
G069P	−	−	−
K001P	−	−	−
K003P	−	−	−
K007P	−	−	No CSF
K008P	−	−	−
K010P	−	−	−
K013P	−	−	−
K014P	−	Cysts	−
K015P	−	−	−
K027P	−	−	−
K028P	−	−	−
K030P	−	−	−
K040P	−	Cysts	(+)
K045P	−	−	−
K053P	−	−	−
K055P	−	−	−
K057P	−	Cysts	−
K060P	−	−	−
K064P	−	−	−
K067P	−	−	−#
K069P	−	−	−
K074P	−	−	−
K075P	−	−	−

(+)* , positive only with the maximum CSF volume (1.2–1.8 ml).

# only 0.2 ml CSF for one PCR available.

Since both PCR and ELISA were used to investigate CSF samples, we next determined the agreement of the two methods. For 48 samples that could be analyzed by both methods, the positive percent agreement was 80.0% (95%CI, 36.0–98.0%) while the negative percent agreement was 90.7% (95%CI, 77.8–96.9%). Expectedly (since the immunoblot may also yield positive results with serum samples from cysticercosis patients without cerebral manifestation), both PCR and ELISA had a low positive percent agreement with the immunoblot (PCR, 18.2%, 95%CI, 9.3–33.2%; ELISA, 11.4%, 95%CI, 4.5–24.4%).

## Discussion

The present study demonstrates a substantial prevalence of cysticercosis amongst PWE in southern Rwanda (the identification of the majority of the NCC cases relied on positive immunoblots together with clinical and epidemiological data thereby allowing the definition of “probable NCC cases” only). A more solid conclusion regarding the NCC prevalence in the study area is hampered by the lack of CT scan results for most of the PWE and all controls; however, the unavailability of neuroimaging at the sites of our study likely represents the factual scenario of many endemic, i.e., resource-poor areas. Nevertheless, we show that NCC may be among the main etiologies of epilepsy in this region, which agrees with the limited data available from sub-Saharan Africa and the reported proportion of NCC among PWE (95% CI: 22.9%–35.5%) [23], [24]. It also supports figures obtained in a small group of 34 Rwandan PWE [12], [25].

In neighboring Burundi, 12% of 103 PWE and 3% of 72 household controls had anticysticercal serum antibodies detected by immunoblot [26], and a subsequent study including 324 PWE and 648 age-matched controls showed a strong link between epilepsy and cysticercosis seropositivity (OR, 3.8; 95% CI, 2.5–5.1) [27]. In Tanzania, anticysticercal but not antitoxocaral antibodies were detected in 15% of 40 PWE [28]. In contrast, a Gambian study could not confirm such an association [29], which is most likely due to differences between the study populations, i.e., 90% of the Gambians but only 5% of the Rwandan population are Muslim and therefore do not eat pork for religious reason.

The present study has several limitations with respect to study design and measurements. Regarding the study design, recruitment was done at a primary, a secondary, and a tertiary health facility to account for potential differences in patient characteristics, but limited to these three facilities because of logistical issues and budget constraints. Undoubtedly, PWE with CT scans were not representative of all PWE seen at these three recruitment sites.

Difficulties in recruiting individuals willing to participate as controls gave rise to a relatively small control group who differed from PWE in several aspects including age and residence. Since the study population is consequently not representative for southern Rwanda, future investigations on NCC in Rwanda should include larger and preferentially matched control groups.

With respect to diagnostic measurements and in analyzing associated factors and clinical manifestations of NCC, we referred to PWE with a positive immunoblot (probable NCC, 70%; definitive, 30%). Two PWE with scolices detected by CT scan were among the immunoblot-negative PWE and this may reflect the sensitivity of the immunoblot (95%, information provided by the manufacturer; 50–92% in a study by Proaño-Narvaez et al. [30]). Notably, grouping based on immunoblot only as done in the present study, or on immunoblot *plus* CT results produced virtually identical results regarding associated factors and clinical characteristics (data not shown). Grouping of NCC including CT scans would have been impractical and imprecise due to the unavailability of a CT scan for the majority of PWE. Nevertheless, CT scans for all participants are desirable for future studies on NCC in this region. These limitations also need to be kept in mind when interpreting the factors associated with NCC (mainly based on immunoblot results).

The odds of NCC increased with age, which may simply reflect increasing exposure over lifetime and a higher proportion of idiopathic or familial epilepsies in children. The association of NCC with reported previous albendazole intake may partially reflect that NCC had already been suspected in some PWE who consequently were treated. Albendazole is the standard drug used for NCC treatment in Rwanda, and it is more effective than praziquantel [31]. Alternatively, albendazole may have been administered either as individual treatment of intestinal helminths or during preventive chemotherapy, e.g., during mass administration. Both scenarios may reflect increased exposure to intestinal helminths (including *T. solium*), which secondarily links with NCC. Unfortunately, details of albendazole treatment were not available. Consumption of pork was another risk factor associated with a positive immunoblot in univariate but not multivariate analysis whereas access to safe drinking water was associated in multivariate analysis with protection against cysticercosis. Valid data on the prevalence of cysticercosis in pigs from Rwanda are missing. More than five decades ago, data from meat inspection estimated the prevalence of porcine cysticercosis as 20% [12]. Certainly, current data are desirable.

With respect to the clinical manifestation of epilepsy, the differences between PWE with positive immunoblot and those with negative immunoblot were remarkable and suggest a substantial contribution of NCC to epilepsy in southern Rwanda. PWE with positive immunoblot were older, had fewer epileptic episodes, and considerably more frequently had late-onset epilepsy than PWE with negative immunoblot. This corresponds with the characteristics of NCC reported from other regions [32].

The diagnosis of NCC in our study was mainly based on the immunoblot results (as a major Del Brutto criterion together with minor and epidemiological criteria), which is the most sensitive and specific tool in cysticercosis serology. One person with epilepsy with NCC-typical lesions and two with suggestive ones were seronegative. This is in line with data demonstrating that the sensitivity of the immunoblot (ranging from 50–92%) depends on the number and type of lesions [30]. In contrast, the CSF ELISA did not contribute substantially to the diagnosis. It was positive in only five PWE four of whom had a positive brain CT scan and subcutaneous cysts, and all five had positive immunoblot results. Interestingly, four of the five CSF specimens from PWE with CSF antibodies were also positive by PCR, suggesting that the sensitivities of both test systems may strongly depend on the intracerebral location of the cysts and thereby on the access of antigen to the subarachnoid space. The greater chance to detect antibodies in serum than in CSF and the higher sensitivity of the immunoblot compared with ELISA have been described previously [30], [33]. Limited sensitivity (54.5%) and specificity (69.2%) of serum ELISA assays have also been reported [34]. While the same study also evaluated an ELISA detecting circulating cysticercal antigen in the serum and reported unsatisfactory results [34], a similar ELISA yielded better results (sensitivity, 100%; specificity, 84%) in a recent study suggesting to include the serum antigen detection as an additional criterion in the Del Brutto criteria [35]. In the present study, *Taenia* eggs were detected in stool samples from only two NCC cases, but stool microscopy is known for its poor sensitivity for the diagnosis of taeniasis [8]. The examination of multiple stool samples might have increased the chance of detection as shown before for intestinal schistosomiasis [36].

PCR has been suggested for the detection of *T. solium*-specific DNA in CSF samples [16]–[18], but no field studies using PCR have been performed thus far. The PCR assay used in the present study showed an overall low sensitivity (16.7%), and three of the eight positive PCR results were only obtained by analyzing the whole volume of the available CSF sample (1.2–1.8 ml). Subsequent studies should therefore determine whether a higher sensitivity can be achieved with larger CSF volumes. Notably, the sensitivity was 43.8% when only the definitive NCC cases were considered. All seven definitive PCR-positive PWE with NCC had typical cerebral lesions while the person with epilepsy and probably NCC with positive PCR result had subcutaneous cysts and a positive immunoblot. Thus, the benefit of the PCR for the definition of NCC cases was limited in our study. This is in contrast to a recent report on a sensitivity of 96% by a different PCR assay evaluated in 121 confirmed NCC cases [17]. However, the specificity of that assay was 80% using samples from an endemic area. Given the high positive predictive value of the PCR assay in our study, i.e., no CSF sample from immunoblot-negative subjects yielded a positive PCR result, PCR assays as the one used in our study could contribute to case definition when CT scans are not feasible but serology is available. In contrast, highly sensitive but less specific PCR assays [17] might be helpful when no serology can be done or in suspicious cases with negative serology.

The present study documents for the first time the public health relevance of cysticercosis in Rwanda and should be followed by more systematic serological investigations because seropositivity within a community may be clustered, e.g., gradients of seroprevalence around tapeworm carriers have been reported [37]. Both a nation-wide health surveillance system and heath education efforts are needed. Additional future control measures might concentrate on interfering with the transmission of *T. solium*. This could be achieved by treatment of tapeworm carriers and/or pigs, health education, improved sanitation, or also by immunizing pigs since a highly effective vaccine has been developed [38] and successfully used in a field trial in Cameroon [39]. Although vaccination certainly is expensive, taking care of cysticercosis patients also is cost-intensive, and recent estimates of the total annual costs due to cysticercosis in West Cameroon amount to more than 10 million Euro with costs per case adding up to 194 Euro [40].

In conclusion and being aware of the aforementioned limitations of our study, NCC appears to be associated with a considerable proportion of late-onset epilepsy in southern Rwanda. Further studies are warranted to ascertain this finding in other parts of the country. The high prevalence of cysticercosis in PWE should influence the management of these patients and argues strongly for establishing facilities of neuroimaging in this area. Furthermore, preventive measures need to be introduced to detect and eliminate the sources of *T. solium* infection.

## Supporting Information

Checklist S1 STROBE Checklist.
(DOC)

Table S1 Baseline characteristics of PWE and controls.
(DOC)

Table S2 Details of neuroimaging results (numbers and types of lesions) of 19 NCC patients for whom CT scan results were available.
(DOC)
