# Supplementary material for: High Prevalence of Cysticercosis in People with Epilepsy in Southern Rwanda
Source: PLoS Negl Trop Dis. 2013 Nov 14;7(11):e2558. doi: 10.1371/journal.pntd.0002558 (PMC3828157; doi:10.1371/journal.pntd.0002558)
Supplement: Table S1 — Baseline characteristics of PWE and controls. (DOC) [file pntd.0002558.s002.doc]

Table S1. Baseline characteristics of PWE and controls

| **Variable** | |  | **PWE** | **Controls** | P |
| --- | --- | --- | --- | --- | --- |
| No. | |  | 215 | 51 |  |
| Age (years; median, range) | | | 23 (10-65) | 40 (15-73) | **< 0.0001** |
| Female sex (%) | |  | 95 (44.2) | 31 (60.8) | **0.03** |
| Location of recruitment (%) | | |  |  |  |
|  | Butare, university hospital | | 43 (20.0) | 23 (45.1) |  |
|  | Kabutare, district hospital | | 85 (39.5) | 15 (29.4) |  |
|  | Gikonko, health center | | 87 (40.5) | 13 (25.5) | **0.0009** |
| Residence, district (%) | | |  |  |  |
|  | Gisagara | | 84 (39.1) | 19 (37.3) |  |
|  | Huye | | 102 (47.0) | 18 (35.3) |  |
|  | Nyamagabe | | 2 (0.9) | 1 (2.0) |  |
|  | Nyanza | | 15 (7.0) | 3 (5.9) |  |
|  | Nyaruguru | | 9 (4.2) | 4 (7.8) |  |
|  | Others | | 4 (1.9) | 6 (11.8) | **0.02** |
| Residence at current location since (%) | | |  |  |  |
|  | Since birth | | 189 (88.3) | 41 (80.4) |  |
|  | >10 years | | 6 (2.8) | 7 (13.7) |  |
|  | 2-10 years | | 13 (6.1) | 2 (3.9) |  |
|  | <2 years | | 6 (2.8) | 1 (2.0) | **0.01** |
| Educational status (%) | | |  |  |  |
|  | No formal education | | 136 (63.3) | 29 (56.9) |  |
|  | Primary school | | 70 (32.6) | 20 (39.2) |  |
|  | Secondary school | | 8 (3.7) | 2 (3.9) |  |
|  | Tertiary school | | 1 (0.5) | 0 | 0.79 |
| Family history of epilepsy (%) | | |  |  |  |
|  | None | | 155 (72.1) | 45 (88.2) |  |
|  | First degree relative(s) | | 30 (14.0) | 2 (3.9) |  |
|  | Other relative(s) | | 30 (14.0) | 4 (7.8) | **0.048** |
| Previous head injury (%) | | |  |  |  |
|  | No | | 180 (83.7) | 49 (96.1) |  |
|  | Yes, with loss of consciousness | | 22 (10.2) | 1 (2.0) |  |
|  | Yes, without loss of consciousness | | 13 (6.0) | 1 (2.0) | 0.07 |
| Previous cerebral vascular event (%) | | | 8/214 (3.7) | 3 (5.9) | 0.49 |
| Previous brain tumor (%) | | | 2 (0.9) | 0 | 0.49 |
| Mental handicap (%) | | | 30 (14.0) | 0 | **0.005** |
| Impaired smelling (%) | | | 2 (0.9) | 0 | 0.49 |
| Impaired visual acuity, visual field (%) | | | 4 (1.9) | 1 (2.0) | 0.96 |
| Impaired facial sensation (%) | | | 1 (0.5) | 0 | 0.63 |
| Impaired hearing or balance (%) | | | 4 (1.9) | 1 (2.0) | 0.96 |
| Impaired voice or speech (%) | | | 10 (4.7) | 0 | 0.12 |
| Muscle tone (%) | |  |  |  |  |
|  | Spastic | | 6 (2.8) | 0 |  |
|  | Flaccid | | 4 (1.9) | 0 | 0.29 |
| Sensation (%) | |  |  |  |  |
|  | Impaired | | 4/214 (1.9) | 1 (2.0) | 0.95 |
| Tendon reflexes (%) | | |  |  |  |
|  | Increased | | 11 (5.1) | 0 |  |
|  | Absent | | 1 (0.5) | 0 | 0.24 |
| Babinski positive (%) | | | 15 (7.0) | 2 (3.9) | 0.42 |
| Romberg positive (%) | | | 9 (4.2) | 0 | 0.14 |
| Skin examination (%) | | |  |  |  |
|  | Subcutaneous cysts | | 9 (4.2) | 0 | 0.20 |
| HIV serology positive (%) | | | 5 (2.3) | 1 (2.0) | 0.87 |
| Previous albendazole treatment (%) | | |  |  |  |
|  | None | | 179 (83.3) | 46 (90.2) |  |
|  | > 6 months ago | | 14 (6.5) | 4 (7.8) |  |
|  | <= 6 months ago | | 22 (10.2) | 1 (2.0) | 0.16 |
| Pigs at home (%) | |  | 125 (58.1) | 23 (45.1) | 0.09 |
| Pigs at neighbours (%) | | | 182 (84.7) | 45 (88.2) | 0.52 |
| Pigs at dwelling (%) | | | 44 (20.6) | 7 (13.7) | 0.27 |
| Pork consumption (%) | | |  |  |  |
|  | Regular | | 62 (28.8) | 16 (31.4) |  |
|  | Seldom | | 111 (51.6) | 21 (41.2) |  |
|  | None | | 42 (19.5) | 14 (27.5) | 0.33 |
| Safe drinking water (%) | | | 32 (14.9) | 7 (13.7) | 0.83 |
| Use of fertilizers (%) | | |  |  |  |
|  | None | | 30 (14.0) | 7 (13.7) |  |
|  | Human | | 3 (1.4) | 0 |  |
|  | Animal | | 182 (84.7) | 44 (86.3) | 0.70 |
| Latrine (%) | |  |  |  |  |
|  | Outdoor | | 209 (97.2) | 50 (98.0) |  |
|  | Indoor | | 5 (2.3) | 0 |  |
|  | None | | 1 (0.5) | 1 (2.0) | 0.30 |
| Cysticercosis immunoblot positive (%) | | | 46/211 (21.8) | 2/50 (4.0) | **0.003** |
