# Supplementary material for: High Prevalence of Cysticercosis in People with Epilepsy in Southern Rwanda
Source: PLoS Negl Trop Dis. 2013 Nov 14;7(11):e2558. doi: 10.1371/journal.pntd.0002558 (PMC3828157; doi:10.1371/journal.pntd.0002558)
Supplement: Table S2 — Details of neuroimaging results (numbers and types of lesions) of 19 NCC patients for whom CT scan results were available. (DOC) [file pntd.0002558.s003.doc]

#### Table S2. Details of neuroimaging results (numbers and types of lesions) of 19 NCC patients for whom CT scan results were available

| Patient | Del Brutto | Parenchymal cysts  (< 2 cm) | Subarachnoid cysts | Vesicular stage/Scolex | Colloidal stage/Edema | Granular stage | Calcifications |
| --- | --- | --- | --- | --- | --- | --- | --- |
| B005P | Definitive |  |  | Scolex detectible^#^ |  |  |  |
| B006P | Definitive | 4 | 4 | 4, scolex detectible | 2 |  |  |
| B011P | Probable | 1 | 1 | - |  | 2 | 10 |
| B014P | Probable | 6 |  | - | 1 | 1 | 4 |
| B016P | Definitive |  |  | Scolex detectible ^#^ |  |  |  |
| B017P | Probable | 1 |  | 1 (no Scolex!) |  |  |  |
| B018P | Definitive | 7 | 3 | 5, scolex detectible | 1 |  | 4 |
| B019P | Definitive | >10 | >5 | >10, scolex detectible | 4 | 4 |  |
| B021P | Definitive |  |  | Scolex detectible ^#^ |  |  |  |
| B026P | Definitive | >10 | 1 | 4, scolex detectible | 5 |  | 4 |
| B032P | Definitive |  |  | Scolex detectible ^#^ |  |  |  |
| B034P | Definitive | >10 | 10 | >10, scolex detectible | 5 | 2 |  |
| B035P | Definitive | >10 | >10 | >10, scolex detectible | 2 |  |  |
| B040P | Definitive | 5 | 5 | 5, scolex detectible | 2 | 2 |  |
| B041P | Definitive | 3 | 2 | 3, scolex detectible |  |  |  |
| B042P | Definitive | 7 |  | 3, scolex detectible | 3 | 1 |  |
| B043P | Definitive | 6 | 4 | 8, scolex detectible | 2 |  |  |
| G027P | Definitive | >10 | >5 | >10, scolex detectible | 2 | 2 |  |
| K087P | Definitive | 5 | 4 | 5, scolex detectible | 2 | 2 |  |

^#^, only information available
